# The Effect of COVID-19 on the Menstrual Cycle: A Systematic Review

**DOI:** 10.3390/jcm11133800

**Published:** 2022-06-30

**Authors:** Vojka Lebar, Antonio Simone Laganà, Vito Chiantera, Tina Kunič, David Lukanović

**Affiliations:** 1 Faculty of Medicine, University of Ljubljana, 1000 Ljubljana, Slovenia; vojka.lebar97@gmail.com; 2 Unit of Gynecologic Oncology, ARNAS “Civico—Di Cristina—Benfratelli”, Department of Health Promotion, Mother and Child Care, Internal Medicine and Medical Specialties (PROMISE), University of Palermo, 90127 Palermo, Italy; antoniosimone.lagana@unipa.it (A.S.L.); vito.chiantera@unipa.it (V.C.); 3 Department of Gynecology, Division of Gynecology and Obstetrics, Ljubljana Medical Center, Zaloška 2, 1000 Ljubljana, Slovenia; tina.kunic@kclj.si

**Keywords:** COVID-19, menstruation disturbances, menstrual cycle

## Abstract

Researchers have been studying COVID-19 from day one, but not much is known about the impact of COVID-19 on the reproductive system, specifically the female reproductive system. There has been substantial anecdotal and media coverage on the effect of COVID-19 on the female reproductive system and changes in the menstrual cycle, but so far available data are not robust enough to draw firm conclusions about the topic. This article was carried out to present already published studies on the correlation between SARS-CoV-2 infection and menstrual cycle changes. A systematic literature search was conducted on the Medline, Scopus, and Cochrane Library databases in accordance with the PRISMA guidelines. Three studies were finally included in the review. The findings of the studies indicate changes in menstrual volume and changes in menstrual cycle length as consequences of SARS-CoV-2 infection; the latter was also the most common menstrual irregularity reported by the included studies. Women have mainly reported decreased menstrual volume and a prolonged cycle. The findings also indicate that the severity of COVID-19 does not play a role in menstrual cycle changes. However, the research on this topic is still too scarce to draw definitive conclusions, and there is a need for further research. The relevant conclusions, which could be drawn only from a well-constructed study, would have a major effect on defining the impact of SARS-CoV-2 infection on the menstrual cycle.

## 1. Introduction

In the past two years, our lives have been significantly affected by COVID-19 and the restrictions the pandemic brought to everyday life. There were 423,437,674 confirmed SARS-CoV-2 infections worldwide as of 21 January 2022 [1]. Researchers have been studying COVID-19 from day one, and there are already robust data about the impact of infection with the SARS-CoV-2 virus on the respiratory [2], circulatory [3], and nervous systems [4]. However, not much is known about the impact of COVID-19 on the reproductive system, specifically the female reproductive system. There has been substantial anecdotal and media coverage on the effect of the COVID-19 pandemic on the female reproductive system and changes in the menstrual cycle [5]; nevertheless, to date the available data are not robust enough to draw firm conclusions about this topic.

In September 2020, 1031 women of reproductive age completed an anonymous digital survey by Phelan et al. as part of an observational study of women’s reproductive health over the course of the pandemic. A total of 46% reported a change in their menstrual cycles since the beginning of the pandemic. The authors found that there was significantly more menorrhagia, dysmenorrhea, and a worsening of premenstrual symptoms than before the pandemic. The length of bleeding and the cycle length were similar to before the pandemic, but with a significantly wider range than before the pandemic. Significant increases in low mood, poor appetite, poor concentration, anxiety, poor sleep, and loneliness were observed [6].

Nevertheless, it is already known that some viral infections correlate with changes in the female reproductive system, such as the duration of the menstrual cycle or the volume of menstruation. For instance, in human immunodeficiency virus (HIV)-positive women, increases in amenorrhea, menstrual cycle intervals lasting more than 6 weeks, and lower rates of premenstrual breast symptoms suggest the possibility of disturbances in menstrual function that do not appear to be attributable to clinically-apparent secondary complications of HIV [7]. Other data have shown a significant association between HIV status and amenorrhea, suggesting that women living with HIV have approximately 1.7 times higher odds of amenorrhea than matched controls without HIV [8]. A study investigating the correlation between hepatitis B virus (HBV)- and hepatitis C virus (HCV)-infection and menstrual cycle changes found the beginning of HBV- and HCV-infection development to be characterized by prolonged, heavy menstruations that were gradually replaced by infrequent and short ones. One out of five patients complained of short and infrequent menstruation (hypomenstrual syndrome), and one out of four complained of the absence of menstruation, including those associated with the onset of early menopause in 6.7% of patients and the development of climacteric syndrome in 6.7% of women. Dysmenorrhea occurred in 11.1% of women [9].

Considering these elements, this article presents published studies about the correlation between COVID-19 and the menstrual cycle and comprehensively assesses the impact of SARS-CoV-2 infection on the menstrual cycle as it is currently understood.

## 2. Materials and Methods

A systematic review was registered in PROSPERO international database (CRD42022339723) and conducted on the Medline, Scopus, and Cochrane Library databases, following the Preferred Reporting Items for Systematic Reviews and Meta-Analyses (PRISMA) Statement, available through the Enhancing the Quality and Transparency Of Health Research (EQUATOR) Network. In Medline, we searched across the medical subject headings (MeSH) using the terms “menstrual cycle” AND “virus diseases”, and on other databases, we searched across all fields for “menstrual cycle” AND “COVID-19”, or “menstrual cycle” AND “COVID*”. Only articles written in English and published between 2020 and 2022 were considered.

Titles and/or abstracts of the studies retrieved using this search strategy and those from additional sources were screened independently by two review authors to identify studies that potentially met the aims of this systematic review. The full texts of these potentially eligible articles were retrieved and independently assessed for eligibility by two other review team members. Any disagreement between the readers regarding the eligibility of particular articles was resolved through discussion with a third (external) reviewer. Two authors independently extracted data from articles about study characteristics and outcomes. Any discrepancies were identified and resolved through discussion (with a third external reviewer where necessary).

Studies that described data on at least one menstrual cycle feature (menstruation length and volume, menstrual cycle length, menstruation regularity and frequency, symptoms of premenstrual syndrome, abnormal bleeding or spotting between normal menstrual periods) and in which participants were defined as having had COVID-19 via positive reverse transcription-quantitative polymerase chain reaction (RT-qPCR)/antigen tests, antibody tests, and/or also suspected cases, were included. Studies not describing the impact of COVID-19 illness itself, but describing the effect of treatments, vaccines, pandemic-related stress, or other lifestyle changes, were excluded. It should also be noted that the focus of this article is only on research articles, although there was no restriction on study design type. Conference presentations and reports were excluded because the goal was to focus on the most carefully evaluated material. The final search was carried out on 22 January 2022.

Due to the nature of the findings, we opted for a narrative synthesis of the results from selected articles.

## 3. Results

**Figure 1:**
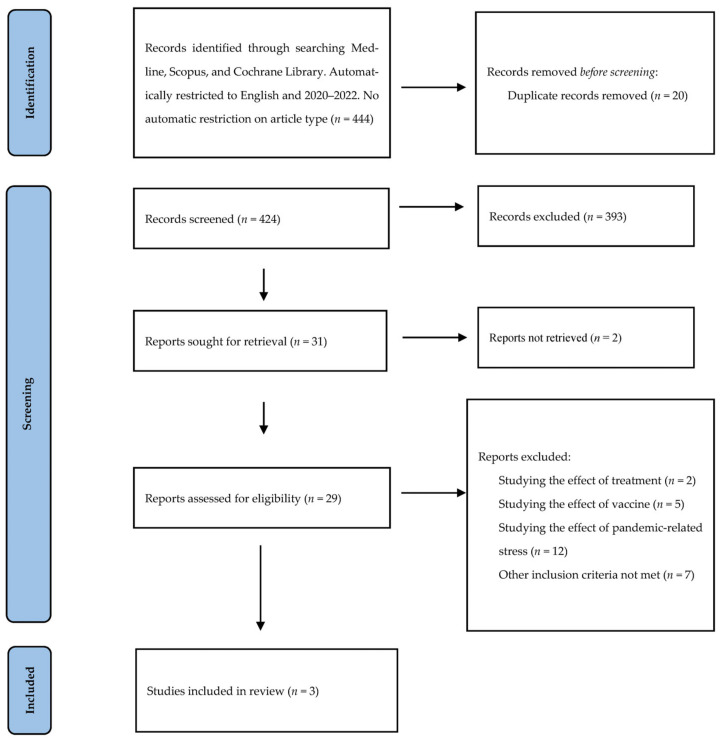
Search strategy and study selection used in this systematic review following the PRISMA protocol.

The aim of this article was to review all the studies that have specifically investigated the impact of COVID-19 illness on menstrual cycle changes and to assess if the correlation between the two is direct, or if menstrual changes are caused by other confounding factors such as stress, vaccines, or a change in lifestyle. Four hundred and forty-four articles were identified and screened at the title and abstract levels (Figure 1).

All but three articles were excluded for any of the following reasons: being duplicates of already identified articles; not assessing menstrual cycle features in the same individuals infected with SARS-CoV-2 over time, or not assessing menstrual cycle features between groups of individuals differentially exposed to COVID-19 (COVID-19 cases and controls). Thus, two cross-sectional studies [10,11] and a cohort study [12] were finally included in this review. All of them included COVID-19-positive patients and studied their menstrual cycle features over a specified period of time (Table 1 and Table 2).

The first study, conducted by Li et al. [10], studied the effects of viral infection on the sex hormone and menstrual changes in women of child-bearing age infected with SARS-CoV-2. It was a single-center, retrospective, cross-sectional study in which they reviewed data from 237 women diagnosed with COVID-19 and hospitalized from 19 January to 1 April 2020. Only women between 18 and 45, not pregnant or lactating, and without menstrual irregularities in the 6 months prior to being infected with COVID-19, were included. There is no information about the use of hormonal contraceptives. COVID-19-positive patients were defined as mild or severe according to signs, symptoms, and complications. There were 147 patients in the mild group and 90 patients in the severe group. Complete menstrual history could be retrieved and reviewed for 177 of the 237 enrolled patients. The information about menstrual cycles was collected by phone for another 2 months after discharge from the hospital. This study found that approximately 20% of the patients had a significant decrease in menstrual volume, without a significant difference between mildly and severely ill patients. Approximately 20% of COVID-19 patients showed prolonged menstrual cycles in comparison with their normal cycles before becoming ill. A smaller number of patients experienced an increase in menstrual volume and/or menstrual cycle shortening. There was no statistically significant difference in any form of menstrual cycle irregularities between mildly and severely ill patients. In comparison with the control group, the menstrual volume and the duration of menstrual cycles changed significantly more for COVID-19-positive women. The analysis of the possible risk factors for menstrual cycle prolongation (age, severity of illness, comorbidities, the presence of complications in other organs, and glucocorticoid treatment) showed that only the presence of complications was associated with menstrual cycle prolongation. In the follow-up period, 84% of the patients returned to their normal menstrual volume and 99% returned to their normal cycle within 1 to 2 months after discharge, suggesting that the menstrual changes due to COVID-19 infection were transient [10].

The second study, by Khan et al. [12], was a prospective, population-based cohort study. It was part of the Arizona CoVHORT study, which is an ongoing study about the long-term consequences of COVID-19 [13], started in May 2020. They included 127 SARS-CoV-2 positive patients who were given COVID-19 symptomology surveys at 6-week intervals. The included patients were 18 to 45 years old and were not or had not recently been pregnant at the time of inclusion. They were asked if they had noticed menstrual cycle changes as an ongoing symptom or if they had noticed new ones related to their COVID-19 illness. Of these, 16% (*n* = 20) reported menstrual cycle changes. The number of days between a positive SARS-CoV-2 test and the last reported menstrual cycle changes varied between 28 and 222 (median 57.5). The most common changes were irregular menstruation (*n* = 12), an increase in premenstrual syndrome symptoms, and infrequent menstruation (*n* = 7). Infrequent menstruation was defined as menstrual periods occurring at intervals longer than 35 days. Irregular menstruation was defined as a significant variation in the length of one’s menstrual period or the time between two periods. The mean age and the mean body mass index (BMI) of the two groups were similar. The percentage of patients of Hispanic ethnicity was higher among patients that had reported menstrual cycle changes (50% vs. 24.3%). The patients that reported menstrual cycle changes were more likely to report a greater number of COVID-19 symptoms (*p* = 0.01). Fatigue, headache, body aches and pains, and shortness of breath were symptoms that were significantly more likely to be reported by patients that reported menstrual cycle changes than patients that did not. The authors were not able to adjust for potential confounding factors. There was no information about how many cycles the changes lasted. There was also no control group or data about the patients’ menstrual cycles before becoming infected [12].

The third study, conducted by Ding et al. [11], was an observational, single-center, cross-sectional study. It was conducted at the start of the COVID-19 pandemic, between 28 January and 8 March 2020, in Wuhan, China. The aim of the study was to investigate the relationship between COVID-19 and ovarian function. A total of 78 female patients with COVID-19, younger than 50 years (the median age was 43.5 years), and without ovarian diseases or an ovarian surgery history were included. None were pregnant or on estrogen-containing contraceptives or menopausal hormone treatments. The patients were asked about their menstrual information in the last 3 months. Of these, 21.79% of the patients were diagnosed as severely ill, 48.0% described a recent mental disorder (e.g., anxiety, depression, or insomnia), 12% had a history of benign gynecological disease, and 36% had undergone gynecological surgery. Severe SARS-CoV-2 infection was defined according to the American Thoracic Society guideline for community-acquired pneumonia [14] and the Guan et al. [15] study of COVID-19 on admission. Patients with more severe COVID-19 cases had higher levels of amenorrhea, higher menstrual volumes, higher levels of irregular periods, and higher levels of menstrual pain compared with non-severe cases, but the differences were not significant [11].

## 4. Discussion

The COVID-19 pandemic has been a theme running through studies and articles in the last two years. Nevertheless, studying one specific aspect of it is difficult because of all the confounding factors that might be interlinked. It is challenging to construct an impeccable study in such circumstances. Several factors of the COVID-19 pandemic must be considered when studying the impact of SARS-CoV-2 infection on the menstrual cycle; in particular, stress, vaccination, COVID-19 therapy, and COVID-19 disease may all play a role.

High stress levels are associated with menstrual irregularities [16]. Because the COVID-19 pandemic has changed peoples’ lives in many aspects (losing loved ones, losing jobs, affecting their health, etc.), it has acted as a stressor, and many researchers believe it has affected menstrual cycles. A recent paper has reported a significant prolongation of menses and heavier bleeding during menses in respondents with a high perceived stress scale (PSS) compared to those with a moderate COVID-19 PSS [17].

There has been substantial media coverage about COVID-19 vaccines causing menstrual cycle irregularities. Up to 2 February 2022, a total of 49,427 menstrual disorders suspected to be caused by vaccination were reported in the UK following the administration of all three COVID-19 vaccines. These included heavier-than-usual periods, delayed periods, and unexpected vaginal bleeding. Approximately 71.8 million COVID-19 vaccine doses were administered to women in the UK up to 2 February 2022 [18]. A study by Edelman et al. found that COVID-19 vaccination was associated with a less than 1-day prolongation of cycle length but not menses length [19]. A recently published study by Laganà et al. [20] reported that approximately 50 to 60% of reproductive-age women that received the first dose of COVID-19 vaccine had menstrual cycle irregularities, regardless of the type of vaccine administered, with a slightly higher (60–70%) occurrence after the second dose. The most common alterations were shorter menstrual cycles, longer cycles, and heavier menstruation than was expected and usual.

Dexamethasone has been important in the treatment of hospitalized COVID-19 patients [21]. It might be a risk factor for menstrual changes in COVID-19 patients, affecting menstrual cycle patterns and blood loss through cortisol [22].

All three studies were conducted in the first half of 2020 during the first wave of the pandemic, so they might be more comparable in terms of virus variants and the impact of a specific wave, since the different waves and variants of the virus had varying impacts on the world population and its health [23,24]. An important aspect of a study is choosing a representative group of participants. Li et al. [10] chose hospitalized COVID-19 patients, which might not be the most representative sample group. Hospital admission itself can be a stressful event for patients and thus affect the menstrual cycle [25]. It also implies a greater severity of disease and other confounding factors that might affect the menstrual cycle. Approximately 7% of people infected with SARS-CoV-2 need hospitalization [26], and hospitalized COVID-19 patients are more likely to be obese, polymorbid, or have metabolic syndrome, which can all cause menstrual irregularities on their own [27,28]. The menstrual data obtained for the studies could be subject to reporting bias because of subjective self-reports of menstrual irregularities. Whereas the first and third studies obtained the menstrual history from medical records, it was not specified how the menstrual history data were gathered. The second study relied on patient reports by asking about changes in the menstrual cycle. The authors of the second study found that patients that reported changes in their menstrual cycle were more likely to report a greater number of COVID-19 symptoms. This could be due to heightened health awareness and could result in reporting bias [29]. Considering all of the information gathered, vaccination could not have played a role in menstrual irregularities in the three studies included in this review [10,11,12] because they had ended by the time the first SARS-CoV-2 vaccine was introduced to the public. Li et al. [10] report assessing for the following risk factors: age, severity of illness, comorbidities, the presence of complications in other organs, and glucocorticoid treatment. Only the presence of complications in other organs was found to be associated with menstrual cycle prolongation. Khan et al. [12] did not adjust for any confounding factors. Ding et al. [11] carried out assessments regarding hormonal changes but not menstrual changes. Treatments the patients received for COVID-19 were not described or controlled for in any of the discussed studies.

## 5. Conclusions

The findings of the studies examined indicate a change in menstrual cycle length and menstrual volume resulting from SARS-CoV-2 infection, mainly a decrease in menstrual volume and a prolongation of the menstrual cycle. They also indicate that the severity of COVID-19 does not play a role in menstrual cycle changes.

The research on this topic is still too scarce to draw definitive conclusions, and there is a need for further research. Indeed, we found only three suitable articles, and all had limitations that should be considered when constructing future studies. Because the SARS-CoV-2 virus is mutating and the restrictions are constantly changing and differ between different countries, it would be beneficial to adequately describe these conditions to make the results of future studies more comparable and more easily put into context. Future research should assess for psychological stress levels, COVID-19 vaccination status, COVID-19 therapy, comorbidities, and other possible confounding factors to distinguish the impact of all of these. It should also strive for more representative, larger sample sizes, including more than just hospitalized COVID-19 patients so that the results can be generalized to a broader population.

The menstrual cycle is an important part of a woman’s life. A normal menstrual cycle is an indicator of good health, and disturbances in the menstrual cycle can indicate underlying conditions. We conclude that future studies are necessary, all of which should include a large sample size, a control group, and an optimal research protocol. The relevant conclusions, which could be drawn only from a well-constructed study, would have a major effect on defining the impact of SARS-CoV-2 infection on the menstrual cycle.

## Figures and Tables

**Table 1:** Summary of the main characteristics of the studies included in the systematic review.

First Author, Year	Type of Study	Comparison	Sample Size	Sample Characteristics	Inclusion Criteria	Exclusion Criteria
Li et al., 2021 [10]	Cross-sectional hospital-based study	COVID-19 patients vs. controls and COVID-19 patients during disease vs. before	177 cases (119 non-severe cases, 58 severe); 91 controls	Average age 36	Women between 18 and 45 years old; confirmed COVID-19	Pregnant or lactating; history of a diagnosis of ovarian dysfunction in the 6 months before onset of disease: manifestation of delayed menses, menstrual irregularities, or earlier menopause; prior hysterectomy or oophorectomy
Khan et al., 2021 [12]	Prospective population-based cohort study	COVID-19 patients that reported a change in menstrual cycle after infection vs. COVID-19 patients that reported no changes	127 (20 participants that reported a change in their menstrual cycle after infection, 107 participants that did not report a change)	Patients that reported menstrual cycle irregularities: mean age 30.5; mean BMI 28.1. Patients that did not report menstrual cycle irregularities: mean age 30.6; mean BMI 27.6	SARS-CoV-2-positive participants that were 18 to 45 years old, identified as women or nonbinary	Currently or recently pregnant, as of January 2020
Ding et al., 2021 [11]	Cross-sectional hospital-based study	Severe vs. non-severe COVID-19 cases	78 (61 non-severe cases, 17 severe)	Median age 43, median BMI 22.7, all had one child or more, 48% had a recent mental disorder, 12% had a history of benign gynecological disease, 36% had undergone gynecological surgery	Female patients of reproductive age and younger than 50	Ovarian diseases or ovarian surgery history; denial of request for blood collection; pregnancy; taking oral or transdermal estrogen-containing products

BMI: body mass index.

**Table 2:** Summary of the main findings of the studies included in the systematic review.

First Author, Year	Main Findings
Li et al., 2021 [10]	Forty-five (25%) patients presented with menstrual volume changes and fifty (28%) patients had menstrual cycle changes, mainly concerning decreased volume (20%) and a prolonged cycle (19%); severely ill patients had more comorbidities than mildly ill patients (34% versus 8%).
Khan et al., 2021 [12]	People that reported changes in their menstrual cycle after SARS-CoV-2 infection reported more COVID-19 symptoms than those that did not. The mean age (30.5 vs. 30.6) and the mean BMI (28.1 vs. 27.0) of the two groups were similar. The percentage of patients of Hispanic ethnicity was higher among the patients that had reported menstrual cycle changes (50% vs. 24.3%).
Ding et al., 2021 [11]	Menstrual status *(p* = 0.55), menstrual volume *(p* = 0.066), phase of menstrual cycle *(p* = 0.58), and dysmenorrhea history *(p* = 0.12) were similar without significant differences between non-severe and severe COVID-19 women

## Data Availability

The study does not report any data.
